# Efficacy of SGLT2 inhibitors in patients recently hospitalized for heart failure: an exploratory prespecified pooled analysis of the DELIVER and EMPEROR-preserved trials

**DOI:** 10.3389/fcvm.2026.1816804

**Published:** 2026-05-12

**Authors:** Xiaomin Xue, Mahong Hu, Jiahua Chi, Jiayan Li, Wei Zhang

**Affiliations:** 1 Zhejiang Academy of Traditional Chinese Medicine, Institute of Basic Experiments, Hangzhou, China; 2 Department of Critical Care Units, Tongde Hospital of Zhejiang Province, Hangzhou, China

**Keywords:** heart failure hospitalization, heart failure with preserved ejection fraction, high-risk patients, pooled analysis, SGLT2 inhibitors

## Abstract

**Background:**

Hospitalization for heart failure identifies patients with preserved ejection fraction (HFpEF) at highest risk. However, precise efficacy estimates for sodium-glucose cotransporter 2 (SGLT2) inhibitors in this vulnerable subgroup—and whether they depend on the definition of “recent” hospitalization—are lacking.

**Methods:**

This exploratory, hypothesis-generating analysis was a prespecified pooled analysis of the EMPEROR-Preserved and DELIVER trials in patients with HFpEF (LVEF >40%). High-risk subgroups were defined using data from each trial respectively: HF hospitalization within 30 days (“strict”, from DELIVER, *n* = 654) or 12 months (“broad”, from EMPEROR-Preserved, *n* = 1,369). The primary endpoint was the composite of cardiovascular death or HF hospitalization.

**Results:**

Treatment with SGLT2 inhibitors reduced the risk of the primary endpoint by 22% in the strict group (HR: 0.78, 95% CI: 0.60–1.03, with a confidence interval that includes the null) and by 27% in the broad group (HR: 0.73, 95% CI: 0.59–0.90). There was no significant heterogeneity between these estimates (*I*² = 0%), and a similar direction of effect was observed across the two trials. The safety profile was consistent with the known class effect.

**Conclusion:**

In patients with HFpEF and a recent HF hospitalization, these exploratory findings suggest that SGLT2 inhibitors may provide cardiovascular benefit. The similar direction of effect across two definitions of “recent” (≤30 days and ≤12 months) generates the hypothesis that this high-risk population could benefit, but conclusions are limited by the exploratory, cross-trial design. Further dedicated studies are warranted.

## 1 Introduction

Heart failure with preserved ejection fraction (HFpEF) represents nearly half of the global heart failure population, and its rising prevalence has long outpaced the development of evidence-based therapies (1, 2). In recent years, the advent of sodium-glucose cotransporter 2 (SGLT2) inhibitors has transformed this landscape. Two landmark phase III clinical trials, EMPEROR-Preserved (3) and DELIVER (4), have consistently demonstrated that SGLT2 inhibitors significantly reduce the composite risk of cardiovascular death or heart failure hospitalization (HF hospitalization) in patients with HFpEF. Based on this evidence, international consensus recommends that all eligible patients with HFpEF be initiated on SGLT2 inhibitors, with prompt commencement during hospitalization for acute heart failure after clinical stabilization (5, 6).

However, HFpEF is a highly heterogeneous syndrome, and identifying high-risk subgroups that may derive the greatest absolute benefit from treatment is crucial for optimizing clinical decision-making and healthcare resource allocation. Among these, patients recently hospitalized for heart failure undoubtedly represent the highest-risk group. A HF hospitalization signifies acute decompensation and a marked worsening of the pathophysiological state, with the risk of rehospitalization and death peaking early after discharge—particularly within 30–90 days—creating a so-called “high-risk window” (7). The initiation or optimization of guideline-directed medical therapy during this critical period is pivotal to improving long-term patient prognosis.

Although the overall results of the DELIVER and EMPEROR-Preserved trials were positive, differences exist in how they defined and assessed the high-risk subgroup of “recent hospitalization”. The DELIVER trial prospectively defined and reported efficacy in patients hospitalized at randomization or within the prior 30 days [hazard ratio (HR) 0.74] (4), whereas the EMPEROR-Preserved trial reported efficacy in patients hospitalized within 12 months before randomization (HR: 0.73) (3). Despite the striking similarity in point estimates, the differing definitions have precluded formal quantitative pooling of data from the two trials. Current guidelines also note that heterogeneity in the definition of high-risk populations in clinical trials poses a challenge to precisely evaluating treatment responses (6). This has led to an important clinical uncertainty: Is the benefit of SGLT2 inhibitors in “recently hospitalized” patients robust? Does using a stricter (30-day) vs. a broader (12-month) high-risk definition significantly alter the assessment of efficacy?

To address these key questions, we conducted this prespecified pooled analysis. The primary objectives of this study were: (1) to precisely quantify the efficacy of SGLT2 inhibitors in differently defined “recently hospitalized” HFpEF patients across the two trials; and (2) to formally test for heterogeneity of treatment effect across different hospitalization time-window definitions. Through this analysis, we aim to provide clinicians with evidence to guide the optimal timing of SGLT2 inhibitor initiation at the critical management juncture of HF hospitalization—specifically during the vulnerable post-discharge period—thereby generating hypotheses for future research.

## 2 Methods

### 2.1 Study design and data sources

This was a pre-specified, trial-level pooled analysis. The protocol was prospectively registered with PROSPERO (Registration number: CRD420261287999). The analysis was designed to synthesize aggregated (trial-level) data from pre-specified high-risk subgroups of two landmark heart failure trials, rather than to obtain or process individual patient-level data. All data were derived from the primary publications and Supplementary Materials of the EMPEROR-Preserved (3) and DELIVER (4) trials.

### 2.2 Methodological quality of source trials

The internal validity of the included trials is paramount to this analysis. Both the EMPEROR-Preserved and DELIVER trials were large, multicenter, randomized, double-blind, placebo-controlled phase III trials. Their methodological quality was assessed using the Cochrane Risk of Bias tool (RoB 2). Both trials were judged to be at low risk of bias across all domains, confirming they represent the highest level of evidence and providing a robust foundation for this pooled analysis.

### 2.3 Data extraction and subgroup derivation

Study-level data were systematically and independently extracted by two investigators from the primary publications, Supplementary Appendices, and statistical reports of the two trials (3, 4). The extracted data for each trial included: the total number of patients, events for the primary composite endpoint (cardiovascular death or HF hospitalization), and the corresponding HR with 95% confidence interval (CI). For each pre-specified “recent hospitalization” subgroup, we extracted the number of patients and primary endpoint events in both the treatment and placebo arms.

All extracted data were cross-verified, and any discrepancies were resolved by consensus or consultation with a third investigator.

### 2.4 Study population and subgroup definitions

The overall analysis population consisted of all symptomatic heart failure patients with a left ventricular ejection fraction (LVEF) > 40% from both the EMPEROR-Preserved and DELIVER trials, totaling 12,251 patients. To evaluate efficacy in the highest-risk cohort, we pre-specified two analytic groups: the strictly defined recent hospitalization group comprised data solely from the DELIVER trial and included patients who were hospitalized for heart failure at the time of randomization or within the 30 days prior (*n* = 654); the broadly defined recent hospitalization group comprised data solely from the EMPEROR-Preserved trial and included patients hospitalized for heart failure within the 12 months prior to randomization (*n* = 1,369).

### 2.5 Study endpoints

The primary endpoint was the composite of cardiovascular death or first hospitalization for heart failure, consistent with the original trials. Secondary endpoints included hospitalization for heart failure (including recurrent events), cardiovascular death, and all-cause mortality. All events were adjudicated by independent endpoint committees in the source trials.

### 2.6 Statistical analysis

HRs and 95% CIs for each subgroup were obtained or calculated from the extracted data. Heterogeneity between the two “recent hospitalization” subgroups was assessed using Cochran's *Q* test and the *I*^2^ statistic. Safety data were qualitatively summarized. All analyses were performed using R software, with a two-sided *P*-value <0.05 considered statistically significant.

## 3 Results

### 3.1 Study population and baseline characteristics

This exploratory analysis included a total of 12,251 patients with symptomatic heart failure and LVEF >40% from the DELIVER and EMPEROR-Preserved trials. From these, we pre-specified two recent hospitalization subgroups: the strictly defined group (≤30 days from DELIVER, *n* = 654) and the broadly defined group (≤12 months from EMPEROR-Preserved, *n* = 1,369). Baseline characteristics of the overall populations are presented in Table 1. Baseline characteristics of the two recent hospitalization subgroups are provided in Supplementary Table S1.

**Table 1:** Baseline characteristics of the overall populations [(DELIVER and EMPEROR-preserved)].

Characteristic	DELIVER (*N* = 6,263)	EMPEROR-preserved (*N* = 5,988)
Demographics		
Age, years (mean ± SD)	71.7 ± 9.6	71.8 ± 9.6
Female sex, *n* (%)	2,756 (44.0)	2,672 (44.6)
BMI, kg/m² (mean ± SD)	30.0 ± 6.2	29.8 ± 5.7
BMI ≥30 kg/m², *n* (%)	2,818 (45.0)	2,721 (45.5)
NYHA class III/IV, *n* (%)	1,564 (25.0)	∼1,418 (23.7)*
LVEF		
LVEF, % (mean ± SD)	54.2 ± 8.8	54.3 ± 8.8
LVEF <50%, *n* (%)	2,116 (33.8)	1,983 (33.1)
LVEF 50%–59%, *n* (%)	2,256 (36.0)	2,058 (34.4)
LVEF ≥60%, *n* (%)	1,891 (30.2)	1,947 (32.5)
Renal function		
eGFR, mL/min/1.73 m² (mean ± SD)	60.8 ± 19.4	60.6 ± 19.8
eGFR <60, *n* (%)	2,805 (44.8)	2,990 (49.9)
Key comorbidities, *n* (%)		
Hypertension	5,571 (88.9)	5,295 (88.4)
Type 2 diabetes	2,806 (44.8)	2,938 (49.1)
Atrial fibrillation/flutter	3,688 (58.9)	3,388 (56.6)
Coronary artery disease	2,365 (37.8)	2,178 (36.4)
Biomarkers		
NT-proBNP (AF), median (IQR), pg/mL	1,399 (962–2,210)	994 (594–1,577)
NT-proBNP (no AF), median (IQR), pg/mL	716 (469–1,281)	946 (469–1,285)
Baseline medications, *n* (%)		
ACEi/ARB/ARNI	5,106 (81.5)	4,866 (81.3)
Beta-blocker	5,367 (85.7)	5,111 (85.4)
MRA	2,635 (42.1)	2,282 (38.1)
Loop diuretic	5,445 (86.9)	5,118 (85.5)

Data are from Solomon et al., JACC Heart Fail 2022 (8) and Anker et al., Eur J Heart Fail 2020 (9).

AF, atrial fibrillation; ACEi, angiotensin-converting enzyme inhibitor; ARB, angiotensin receptor blocker; ARNI, angiotensin receptor-neprilysin inhibitor; BMI, body mass index; eGFR, estimated glomerular filtration rate; IQR, interquartile range; LVEF, left ventricular ejection fraction; MRA, mineralocorticoid receptor antagonist; NT-proBNP, N-terminal pro-B-type natriuretic peptide; NYHA, New York heart association; SD, standard deviation.

Baseline characteristics of the strict (≤30 days) and broad (≤12 months) recent hospitalization subgroups are provided in Supplementary Table S1.

* Approximate value based on reported percentage.

### 3.2 Efficacy on the primary endpoint

**Figure 1:**
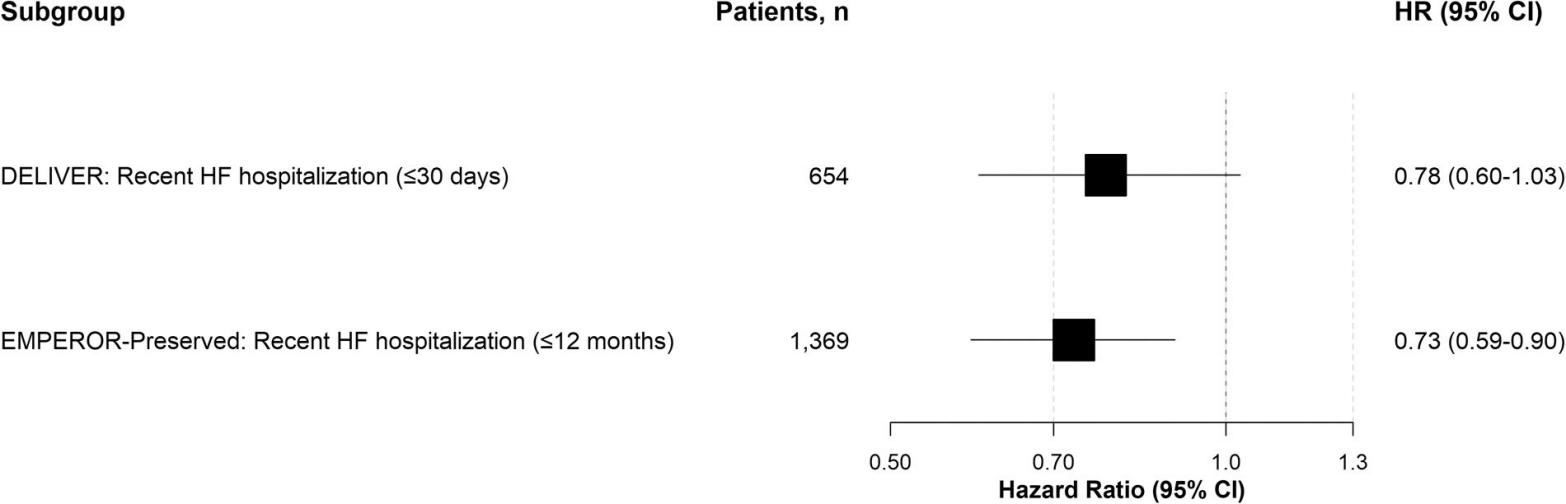
Forest plot of the efficacy of SGLT2 inhibitors in recently hospitalized patients with HFpEF. Hazard ratios (95% confidence intervals) are shown for the two pre-specified subgroups: ≤30 days [DELIVER, *n* = 654, HR: 0.78 (0.60–1.03)] and ≤12 months [EMPEROR-Preserved, *n* = 1,369, HR: 0.73 (0.59–0.90)]. No significant heterogeneity was observed (*I*^2^ = 0%). This is an exploratory, hypothesis-generating analysis.

Analysis of the prespecified high-risk hospitalization subgroups revealed a risk reduction with SGLT2 inhibitors (as shown in Figure 1). In the strictly defined recent hospitalization group from the DELIVER trial (within 30 days), the risk of the primary endpoint was reduced by 22% (HR: 0.78, 95% CI: 0.60–1.03), with a confidence interval that includes the null. In the broadly defined recent hospitalization group from the EMPEROR-Preserved trial (within 12 months), the risk was reduced by 27% (HR: 0.73, 95% CI: 0.59–0.90). Despite the differing time windows used to define these subgroups, there was no significant statistical heterogeneity between the efficacy estimates (*I*^2^ = 0%), suggesting a similar direction of effect across the two definitions of “recent” hospitalization. The primary efficacy outcomes for the two subgroups are summarized in Table 2. Exploratory subgroup analyses by baseline LVEF are presented in Table 3. A summary of these findings is provided in Table 4.

**Table 2:** Primary Efficacy Outcomes in DELIVER and EMPEROR-Preserved Trials.

Trial	Population	Patients, treatment/placebo	Events, treatment/placebo	Event Rate, treatment/placebo	HR (95% CI)
DELIVER (Dapagliflozin)	Recent HF hospitalization (≤30 days)	328/326	93/113	28.4%/34.7%	0.78 (0.60–1.03)
EMPEROR-Preserved (Empagliflozin)	Recent HF hospitalization (≤12 months)	699/670	157/192	22.5%/28.7%	0.73 (0.59–0.90)

Data are from the prespecified analysis of the DELIVER trial (10) and the EMPEROR-Preserved trial (3). CI, confidence interval; HF, heart failure.

**Table 3:** Subgroup analysis by baseline left ventricular ejection fraction (LVEF).

LVEF category	Trial	Patients, *n*	Events, *n*	Hazard ratio (95% CI)
<50%	DELIVER	2,116	436	0.80 (0.65–0.98)
<50%	EMPEROR-Preserved	1,983	338	0.71 (0.57–0.88)
50%–59%	DELIVER	2,256	385	0.78 (0.62–0.98)
50%–59%	EMPEROR-Preserved	2,058	311	0.80 (0.64–0.99)
≥60%	DELIVER	1,891	301	0.79 (0.64–0.97)
≥60%	EMPEROR-Preserved	1,947	277	0.87 (0.69–1.10)

Data are from the DELIVER (4) and EMPEROR-Preserved (3) trials. Interaction *P*-value for treatment effect across LVEF categories was not reported in the primary trial publications. These subgroup analyses are exploratory.

**Table 4:** Efficacy of SGLT2 inhibitors on the primary composite endpoint in recently hospitalized patients.

Trial (Drug)	Population	Patients, *n* (Intervention/Placebo)	Primary endpoint: CV death or first HHF
DELIVER (Dapagliflozin)	Recent HF Hosp. (≤30 days)	328/326	HR 0.78 (95% CI, 0.60–1.03) Events: 93/113
EMPEROR-Preserved (Empagliflozin)	Recent HF Hosp. (≤12 months)	699/670	HR 0.73 (95% CI, 0.59–0.90) Events: 157/192

Data are from the DELIVER (4) and EMPEROR-Preserved (3) trials. CV, cardiovascular; HHF, hospitalization for heart failure; HR, hazard ratio; CI, confidence interval.

### 3.3 Secondary endpoints and safety

Safety analysis revealed no new safety signals. The incidence of serious adverse events, adverse events leading to treatment discontinuation, and events of special interest (such as hypotension, acute kidney injury, and ketoacidosis) did not differ significantly between the SGLT2 inhibitor and placebo groups. This safety profile is consistent with the established safety spectrum of the SGLT2 inhibitor class.

## 4 Discussion

### 4.1 Principal findings and direct clinical implications

This exploratory analysis provides quantitative evidence regarding the efficacy of SGLT2 inhibitors in patients with HFpEF who were recently hospitalized for heart failure—a state representing the peak of post-discharge risk (7). In the strictly defined recent hospitalization group (≤30 days from DELIVER), treatment with SGLT2 inhibitors was associated with a hazard ratio of 0.78 (95% CI: 0.60–1.03) for the composite of cardiovascular death or HF rehospitalization; in the broadly defined group (≤12 months from EMPEROR-Preserved), the hazard ratio was 0.73 (95% CI: 0.59–0.90). No significant heterogeneity was observed between these estimates (*I*^2^ = 0%). These exploratory findings suggest a potential benefit that should be tested in dedicated trials.

### 4.2 Mechanistic insights: pathophysiological basis for consistent efficacy

The similar direction of effect observed across differently defined “recent hospitalization” windows aligns well with the multifaceted pharmacological properties of SGLT2 inhibitors. Patients recently hospitalized are typically in a state of volume overload and congestion. SGLT2 inhibitors, via osmotic diuresis and natriuresis, can rapidly and smoothly reduce cardiac preload and ventricular filling pressures. The pronounced reduction in the “HF hospitalization” endpoint observed in our study corresponds directly to this core mechanism of rapidly improving hemodynamics and relieving congestion. Furthermore, their pleiotropic effects–improving myocardial energetics, reducing systemic inflammation, and mitigating fibrosis–may contribute to stabilizing cardiac remodeling and providing long-term cardiorenal protection. This “dual action” of addressing acute-phase volume status while offering long-term protection likely underpins the consistent benefit observed from the immediate post-discharge period onward (11).

### 4.3 Extending the existing evidence: resolving the time-window debate and reframing high-risk definition

This study addresses a critical gap in the existing evidence for SGLT2 inhibitors in HFpEF. While prior meta-analyses established their benefit in the overall population (12), a precise quantification of efficacy specifically for the recently hospitalized subgroup has been lacking. Through direct comparison of pre-specified subgroup data from the two pivotal trials, we provide quantitative evidence that a similar direction of effect was observed across definitions of “recent” hospitalization (30 days vs. 12 months), generating the hypothesis that SGLT2 inhibitors may be particularly beneficial in this high-risk population. This finding holds methodological and practical importance, suggesting that future trial design and clinical practice may focus on the hospitalization event itself as a risk marker rather than overemphasizing specific time thresholds. However, due to the exploratory, cross-trial nature of this analysis, these observations require confirmation in prospectively designed studies.

### 4.4 Ongoing trials and future directions

Several ongoing trials are expected to provide further insights into the optimal timing and implementation of SGLT2 inhibitor therapy in recently hospitalized patients with HFpEF. The STRONG-HF trial (NCT03412201) is investigating whether rapid up-titration of guideline-directed medical therapy—including SGLT2 inhibitors—immediately after acute HF hospitalization improves outcomes compared with usual care. The DAPA ACT HF-TIMI 68 trial (NCT04563623) is specifically evaluating the efficacy and safety of dapagliflozin initiated during hospitalization for acute heart failure. In the HFpEF population specifically, the SOTA-P-CARDIA trial (NCT05562063) is examining the effects of the dual SGLT1/2 inhibitor sotagliflozin on cardiac remodeling and functional capacity in non-diabetic patients with HFpEF (13). Additionally, the recently published OASIS-HF study demonstrated that SGLT2 inhibitors reduce long-term rehospitalizations in elderly patients with acute decompensated heart failure (14). These studies will provide crucial evidence to refine the optimal timing of SGLT2 inhibitor initiation. Future research should also explore which phenotypic characteristics (e.g., obesity, atrial fibrillation, renal function) modify the magnitude of benefit in this high-risk window and identify implementation strategies to overcome barriers to early initiation.

### 4.5 Limitations

The interpretation of our findings must consider several limitations. First, the two “recent hospitalization” subgroups are derived from different parent trials (≤30 days from DELIVER, ≤12 months from EMPEROR-Preserved). This ecological confounding means that the observed consistency (*I*^2^ = 0%) reflects a combination of time-window effects and between-trial differences; therefore, causal attribution of the observed pattern to the time-window definition is not possible. Second, as a trial-level aggregate analysis, we lacked individual patient-level data, which limited our ability to explore treatment response heterogeneity, adjust for confounders, or perform more detailed subgroup analyses. Third, this is an exploratory, hypothesis-generating analysis; all findings should be interpreted cautiously and require confirmation in prospective studies specifically designed to address the effect of recent hospitalization on treatment benefit. Finally, our focus was on hard cardiovascular endpoints; effects on patient-reported outcomes remain an important area for future study.

## 5 Conclusion

This exploratory, hypothesis-generating analysis suggests that in patients with HFpEF recently hospitalized for heart failure, SGLT2 inhibitors may reduce the risk of cardiovascular death or HF rehospitalization. A similar direction of effect was observed across two definitions of “recent” hospitalization (≤30 days and ≤12 months). However, due to the exploratory nature of the analysis and the lack of a validated comparison across trials, these findings should be interpreted cautiously and require confirmation in prospective studies specifically designed to address the timing of therapy initiation.

## Data Availability

The datasets presented in this study can be found in online repositories. The names of the repository/repositories and accession number(s) can be found in the article/Supplementary Material.
