# Supplementary material for: Efficacy of SGLT2 inhibitors in patients recently hospitalized for heart failure: an exploratory prespecified pooled analysis of the DELIVER and EMPEROR-preserved trials
Source: Front Cardiovasc Med. 2026 May 12;13:1816804. doi: 10.3389/fcvm.2026.1816804 (PMC13201419; doi:10.3389/fcvm.2026.1816804)
Supplement: Supplementary file 1 [file table1.docx]

### Supplementary Table S1. Baseline Characteristics of the Two Recent Hospitalization Subgroups

| Characteristic | Strict Recent-HF (≤30 days, DELIVER, n=654) | Broad Recent-HF (≤12 months, EMPEROR-Preserved, n=1,369) |
| --- | --- | --- |
| ****Demographics**** |  |  |
| Age, years (mean ± SD) | 72.5 ± 9.8* | 72.0 ± 9.7* |
| Female sex, n (%) | 280 (42.8)* | 580 (42.4)* |
| BMI, kg/m² (mean ± SD) | 30.3 ± 6.4* | 29.9 ± 5.8* |
| BMI ≥30 kg/m², n (%) | 301 (46.0)* | 624 (45.6)* |
| NYHA class III/IV, n (%) | 169 (25.8)* | 329 (24.0)* |
| ****LVEF**** |  |  |
| LVEF, % (mean ± SD) | 53.8 ± 8.9* | 53.9 ± 8.8* |
| LVEF <50%, n (%) | 223 (34.1)* | 453 (33.1)* |
| LVEF 50–59%, n (%) | 235 (35.9)* | 470 (34.3)* |
| LVEF ≥60%, n (%) | 196 (30.0)* | 446 (32.6)* |
| ****Renal function**** |  |  |
| eGFR, mL/min/1.73 m² (mean ± SD) | 58.3 ± 20.1* | 58.9 ± 19.8* |
| eGFR <60, n (%) | 319 (48.8)* | 712 (52.0)* |
| ****Key comorbidities, n (%)**** |  |  |
| Hypertension | 585 (89.4)* | 1,215 (88.8)* |
| Type 2 diabetes | 380 (58.1)* | 750 (54.8)* |
| Atrial fibrillation/flutter | 410 (62.7)* | 795 (58.1)* |
| Coronary artery disease | 265 (40.5)* | 520 (38.0)* |
| ****Biomarkers**** |  |  |
| NT‑proBNP (AF), median (IQR), pg/mL | NR | 1,420 (890–2,100)* |
| NT‑proBNP (no AF), median (IQR), pg/mL | NR | 1,380 (860–2,230)* |
| ****Baseline medications, n (%)**** |  |  |
| ACEi/ARB/ARNI | 525 (80.3)* | 1,105 (80.7)* |
| Beta‑blocker | 562 (85.9)* | 1,172 (85.6)* |
| MRA | 294 (45.0)* | 540 (39.4)* |
| Loop diuretic | 615 (94.0)* | 1,280 (93.5)* |

**Notes:** * Values are estimated based on overall trial data assuming similar distribution within subgroups, as exact subgroup‑specific data were not reported in the source publications (see Supplementary Methods for details).

NR = not reported in the source trial for this subgroup.

Data for the strict recent hospitalization group (≤30 days) are from Cunningham et al., J Am Coll Cardiol. 2022;80(14):1302‑10 (prespecified analysis of DELIVER trial), with additional variables estimated where not directly reported.

Data for the broad recent hospitalization group (≤12 months) are from Anker et al., Eur J Heart Fail. 2020;22(12):2383‑92 (baseline characteristics of EMPEROR‑Preserved patients hospitalized within 12 months), with additional variables estimated where not directly reported.

All values with an asterisk (*) are estimated. Values marked NR were not reported in the source trial for this subgroup.

Abbreviations as in Table 1.
